# Classification of Rota-Baxter operators on semigroup algebras of order two and three

**DOI:** 10.1080/00927872.2018.1552278

**Published:** 2019-03-04

**Authors:** Shanghua Zheng, Li Guo, Markus Rosenkranz

**Affiliations:** a Department of Mathematics, Jiangxi Normal University, Nanchang, Jiangxi, China;; b Department of Mathematics and Computer Science, Rutgers University, Newark, New Jersey, USA;; c Research Institute for Symbolic Computation (RISC), Johannes Kepler University, Linz, Austria

**Keywords:** Matrix, Mathematica, Rota-Baxter algebra, semigroup, semigroup algebra, 16W99, 16S36, 20M25, 16Z05

## Abstract

In this paper, we determine all the Rota-Baxter operators of weight zero on semigroup algebras of order two and three with the help of computer algebra. We determine the matrices for these Rota-Baxter operators by directly solving the defining equations of the operators. We also produce a Mathematica procedure to predict and verify these solutions.

## 1. Introduction

Rota-Baxter operators arose from the probability study of G. Baxter in 1960 [7], defined by the operator identity

(1) P(x)P(y)=P(xP(y))+P(P(x)y)+λP(xy),

where *λ* is a fixed scalar called the weight. When *λ* = 0, the operator is a natural algebraic generalization of the integral operator. In the 1960s and 70 s, these operators attracted attention from well-known analysts such as Atkinson [2] and combinatorialists such as Cartier and Rota [10, 39, 40]. In the 1980s these operators were studied in integrable systems as the operator form of the classical Yang-Baxter equations [41], named after the well-known physicists C.N. Yang and R.J. Baxter. Since the late 1990s, the study of Rota-Baxter operators has made great progress both in theory and in applications in combinatorics, number theory, operads, boundary value problems and mathematical physics [3–6, 9, 11, 17, 20–22, 38].

Rota-Baxter algebras arising naturally from applications as well as theoretical investigations (e.g. free Rota-Baxter algebras [16, 21]) are mostly infinite dimensional. To study finite dimensional Rota-Baxter algebras in general, it is useful to start with low dimensional Rota-Baxter algebras. Even there computations are quite complicated. In recent years, some progress regarding such computations has been achieved, with applications to pre-Lie algebras, dendriform algebras and the classical Yang-Baxter equation [1, 28, 35, 43]. In this paper, we study Rota-Baxter operators on a class of low dimensional algebras, namely semigroup algebras for small order semigroups.

Semigroup algebras, a natural generalization of group algebras, form an important class of associative algebras arising from semigroups [34]. The representation of semigroups leads to a semigroup algebra satisfying polynomial identities. In this regards, Rota-Baxter operators on a semigroup algebra can be regarded as an operated semigroup algebra satisfying an operator identity. It has been shown that every finite dimensional algebra of finite representation type over an algebraically closed field is a contracted semigroup algebra. Recently, semigroup algebras have experienced rapid development on the theoretical side [13, 14, 24, 25] as well as in applications to representation theory, cohomology, geometric group theory, topology, combinatorics, algebraic geometry, and number theory [8, 19, 26, 27, 30–33, 42]. Thus studying semigroup algebras in their canonical basis has a significance of its own.

In this paper, we classify all Rota-Baxter operators on semigroup algebras of order 2 and 3. Through studying Rota-Baxter operators on low dimensional semigroup algebras, we hope to find patterns for the study of Rota-Baxter operators on general semigroup algebras. See Section 6 for further details. Rota-Baxter operators on associative algebras of dimension 2 and 3 have been determined up to isomorphism in [28]. Here we focus on the particular presentation of such an algebra in terms of the canonical semigroup algebra basis, because of the aforementioned importance of using such a basis. Indeed, as one notices by comparing the classifications given here with the ones in [28], the resulting Rota-Baxter operators take a very different form.

Because of the complex nature of Rota-Baxter operators, determining their classification by hand is challenging even for low dimensional algebras, as observed in [1, 28, 43]. In such a case, computer algebra provides an indispensable aid for both predicting and verifying these operators. Nevertheless, for ensuring theoretical accuracy, it is still necessary to carry out a rigorous proof of the classification. In Section 2, we start by developing the general setup of the equations that serve as the necessary and sufficient conditions characterizing a Rota-Baxter operator on a semigroup algebra. We then provide the Mathematica procedure that has helped us in solving the classification problem. In Section 3, we classify all Rota-Baxter operators on semigroup algebras of order 2. For Rota-Baxter operators on semigroup algebras of order 3, we carry out the classification in two sections, with Section 4 for commutative semigroup algebras and Section 5 for noncommutative semigroup algebras. We end the paper with some conclusion remarks.

## 2. The general setup and the computer algebra procedure

In this section, we first formulate the general setup for determining Rota-Baxter operators of weight zero on a semigroup algebra. We then implement this setup in Mathematica to obtain a procedure that helped us to obtain classifications of Rota-Baxter operators on semigroup algebras of order two and three.

### 2.1. The general setup

In this subsection, we give the general setup of Rota-Baxter operators on a semigroup algebra in matrix form. Let *S* be a finite semigroup with multiplication · that we often suppress. Thus $S=\{e_{1},\ldots ,e_{n}\}$. Let k be a commutative unitary ring and let

(2) $$k[S]:=\sum_{m=1}^{n}ke_{m}=\left\{\sum_{m=1}^{n}a_{m}e_{m}|a_{m}\in k,1⩽m⩽n\right\}$$

denote the semigroup algebra of *S*. The order *n* of the semigroup *S* is also said to be the **order** of the semigroup algebra k[S].

Let P:k[S]→k[S] be a Rota-Baxter operator of weight zero. Since *P* is k-linear, we have

(3) $$\left(\begin{array}{c} P(e_{1})\\ P(e_{2})\\ \cdots \\ P(e_{n}) \end{array}\right)=\left(\begin{array}{ccc} c_{11} & \cdots & c_{1n}\\ c_{21} & \cdots & c_{2n}\\ \cdots & \cdots & \cdots \\ c_{n1} & \cdots & c_{nn} \end{array}\right)\left(\begin{array}{c} e_{1}\\ e_{2}\\ \cdots \\ e_{n} \end{array}\right)\;(c_{ij}\in k,1⩽i,j⩽n).$$

The matrix $C:=C_{P}:={(c_{ij})}_{1⩽i,j⩽n}$ is called the **matrix of P**. Further, *P* is a Rota-Baxter operator if and only if

(4) $$P(e_{i})P(e_{j})=P(P(e_{i})e_{j}+e_{i}P(e_{j}))\;(1⩽i,j⩽n).$$

Let the Cayley (multiplication) table of the semigroup *S* be given by

(5) $$e_{k}\cdot e_{\ell }=\sum_{m=1}^{n}r_{k\ell }^{m}e_{m}\;(1⩽k,\ell ⩽n),$$

where $r_{k\ell }^{m}\in \{0,1\}$. Then we have

$$P(e_{i})P(e_{j})=\sum_{k=1}^{n}\sum_{\ell =1}^{n}c_{ik}c_{j\ell }e_{k}e_{\ell }=\sum_{m=1}^{n}\sum_{k=1}^{n}\sum_{\ell =1}^{n}r_{k\ell }^{m}c_{ik}c_{j\ell }e_{m}$$

and

$$\begin{array}{l} P(P(e_{i})e_{j}+e_{i}P(e_{j}))=\sum_{k=1}^{n}c_{ik}P(e_{k}e_{j})+\sum_{\ell =1}^{n}c_{j\ell }P(e_{i}e_{\ell })\\ =\sum_{k=1}^{n}\sum_{m=1}^{n}r_{kj}^{m}c_{ik}P(e_{m})+\sum_{\ell =1}^{n}\sum_{m=1}^{n}r_{i\ell }^{m}c_{j\ell }P(e_{m})\\ =\sum_{k=1}^{n}\sum_{m=1}^{n}r_{kj}^{m}c_{ik}(\sum_{\ell =1}^{n}c_{m\ell }e_{\ell })+\sum_{k=1}^{n}\sum_{m=1}^{n}r_{ik}^{m}c_{jk}(\sum_{\ell =1}^{n}c_{m\ell }e_{\ell })\\ =\sum_{m=1}^{n}\sum_{\ell =1}^{n}\sum_{k=1}^{n}\left(r_{kj}^{\ell }c_{ik}+r_{ik}^{\ell }c_{jk}\right)c_{\ell m}e_{m}. \end{array}$$

Thus we obtain

Theorem 2.1. *Let* $S=\{e_{1},\ldots ,e_{n}\}$ *be a semigroup with its Cayley table given by Eq. (5). Let* k *be a commutative unitary ring and let* P:k[S]→k[S] *be a linear operator with matrix* $C:=C_{P}={\left(c_{ij}\right)}_{1⩽i,j⩽n}$*. Then P is a Rota-Baxter operator of weight zero on* k[S] *if and only if the following equations hold.*

(6) $$\sum_{\ell =1}^{n}\sum_{k=1}^{n}r_{k\ell }^{m}c_{ik}c_{j\ell }=\sum_{\ell =1}^{n}\sum_{k=1}^{n}\left(r_{kj}^{\ell }c_{ik}+r_{ik}^{\ell }c_{jk}\right)c_{\ell m}\;(1⩽i,j,m⩽n).$$

*We will determine the matrices C_P_ for all Rota-Baxter operators P on* k[S] *of order two or three.*

### 2.2. The Mathematica procedure

In this subsection, we describe the computer algebra procedure (implemented in *Mathematica*) for computing the Rota-Baxter operators on semigroup algebras of semigroup of order 3, listed in Tables 1 and 2. This procedure serves both for guiding and verifying the manual proofs of the classification theorems carried out in later sections of the paper.

**Table 1.** Table of commutative semigroups of order 3.

CS of order 3	CS of order 3	CS of order 3	CS of order 3
$CS(1):=\begin{array}{cccc} \cdot & e_{1} & e_{2} & e_{3}\\ e_{1} & e_{1} & e_{1} & e_{1}\\ e_{2} & e_{1} & e_{1} & e_{1}\\ e_{3} & e_{1} & e_{1} & e_{1} \end{array}$	$CS(2):=\begin{array}{cccc} \cdot & e_{1} & e_{2} & e_{3}\\ e_{1} & e_{1} & e_{1} & e_{1}\\ e_{2} & e_{1} & e_{1} & e_{1}\\ e_{3} & e_{1} & e_{1} & e_{2} \end{array}$	$CS(3):=\begin{array}{cccc} \cdot & e_{1} & e_{2} & e_{3}\\ e_{1} & e_{1} & e_{1} & e_{1}\\ e_{2} & e_{1} & e_{2} & e_{1}\\ e_{3} & e_{1} & e_{1} & e_{1} \end{array}$	$CS(4):=\begin{array}{cccc} \cdot & e_{1} & e_{2} & e_{3}\\ e_{1} & e_{1} & e_{1} & e_{1}\\ e_{2} & e_{1} & e_{2} & e_{1}\\ e_{3} & e_{1} & e_{1} & e_{3} \end{array}$
$CS(5):=\begin{array}{cccc} \cdot & e_{1} & e_{2} & e_{3}\\ e_{1} & e_{1} & e_{1} & e_{1}\\ e_{2} & e_{1} & e_{2} & e_{2}\\ e_{3} & e_{1} & e_{2} & e_{2} \end{array}$	$CS(6):=\begin{array}{cccc} \cdot & e_{1} & e_{2} & e_{3}\\ e_{1} & e_{1} & e_{1} & e_{1}\\ e_{2} & e_{1} & e_{2} & e_{2}\\ e_{3} & e_{1} & e_{2} & e_{3} \end{array}$	$CS(7):=\begin{array}{cccc} \cdot & e_{1} & e_{2} & e_{3}\\ e_{1} & e_{1} & e_{1} & e_{1}\\ e_{2} & e_{1} & e_{2} & e_{3}\\ e_{3} & e_{1} & e_{3} & e_{1} \end{array}$	$CS(8):=\begin{array}{cccc} \cdot & e_{1} & e_{2} & e_{3}\\ e_{1} & e_{1} & e_{1} & e_{1}\\ e_{2} & e_{1} & e_{2} & e_{3}\\ e_{3} & e_{1} & e_{3} & e_{2} \end{array}$
$CS(9):=\begin{array}{cccc} \cdot & e_{1} & e_{2} & e_{3}\\ e_{1} & e_{1} & e_{1} & e_{3}\\ e_{2} & e_{1} & e_{1} & e_{3}\\ e_{3} & e_{3} & e_{3} & e_{1} \end{array}$	$CS(10):=\begin{array}{cccc} \cdot & e_{1} & e_{2} & e_{3}\\ e_{1} & e_{1} & e_{1} & e_{3}\\ e_{2} & e_{1} & e_{2} & e_{3}\\ e_{3} & e_{3} & e_{3} & e_{1} \end{array}$	$CS(11):=\begin{array}{cccc} \cdot & e_{1} & e_{2} & e_{3}\\ e_{1} & e_{1} & e_{2} & e_{2}\\ e_{2} & e_{2} & e_{1} & e_{1}\\ e_{3} & e_{2} & e_{1} & e_{1} \end{array}$	$CS(12):=\begin{array}{cccc} \cdot & e_{1} & e_{2} & e_{3}\\ e_{1} & e_{1} & e_{2} & e_{3}\\ e_{2} & e_{2} & e_{3} & e_{1}\\ e_{3} & e_{3} & e_{1} & e_{2} \end{array}$

**Table 2.** Cayley table of noncommutative semigroups of order 3.

$NCS(1):=\begin{array}{cccc} \cdot & e_{1} & e_{2} & e_{3}\\ e_{1} & e_{1} & e_{1} & e_{1}\\ e_{2} & e_{1} & e_{2} & e_{1}\\ e_{3} & e_{1} & e_{3} & e_{1} \end{array}$	$NCS(2):=\begin{array}{cccc} \cdot & e_{1} & e_{2} & e_{3}\\ e_{1} & e_{1} & e_{1} & e_{1}\\ e_{2} & e_{1} & e_{2} & e_{1}\\ e_{3} & e_{3} & e_{3} & e_{3} \end{array}$	$NCS(3):=\begin{array}{cccc} \cdot & e_{1} & e_{2} & e_{3}\\ e_{1} & e_{1} & e_{1} & e_{1}\\ e_{2} & e_{1} & e_{2} & e_{2}\\ e_{3} & e_{1} & e_{3} & e_{3} \end{array}$
$NCS(4):=\begin{array}{cccc} \cdot & e_{1} & e_{2} & e_{3}\\ e_{1} & e_{1} & e_{1} & e_{1}\\ e_{2} & e_{2} & e_{2} & e_{2}\\ e_{3} & e_{1} & e_{1} & e_{1} \end{array}$	$NCS(5):=\begin{array}{cccc} \cdot & e_{1} & e_{2} & e_{3}\\ e_{1} & e_{1} & e_{1} & e_{1}\\ e_{2} & e_{2} & e_{2} & e_{2}\\ e_{3} & e_{3} & e_{3} & e_{3} \end{array}$	$NCS(6):=\begin{array}{cccc} \cdot & e_{1} & e_{2} & e_{3}\\ e_{1} & e_{1} & e_{1} & e_{1}\\ e_{2} & e_{1} & e_{2} & e_{3}\\ e_{3} & e_{3} & e_{3} & e_{3} \end{array}$

**Figure 1.**
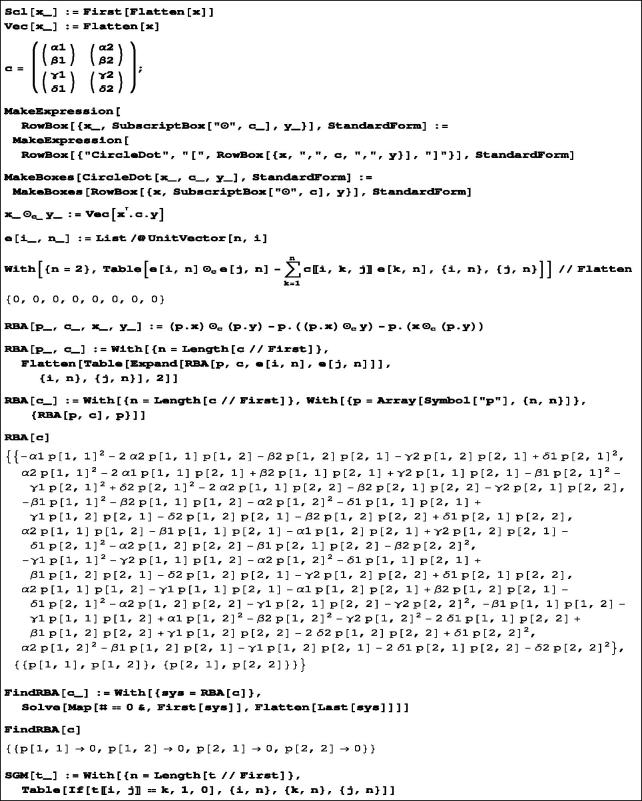
The procedure for computing Rota-Baxter operators on semigroup algebras.

The Mathematica code and accompanying syntax definitions are given in Figure 1. The function RBA with four arguments creates Eq. (6) for a fixed pair of elements, which are then instantiated by all generator pairs. For added clarity, we have also displayed the general form of these equations for the generic 2 × 2 Cayley table defined at the beginning. The main function for determining Rota-Baxter operators is FindRBO, which works by solving the equations created by RBA. For converting a given Cayley table to the structure constants $r_{kl}^{m}$ used in Eq. (6), the function SGM is employed.

**Figure 2.**
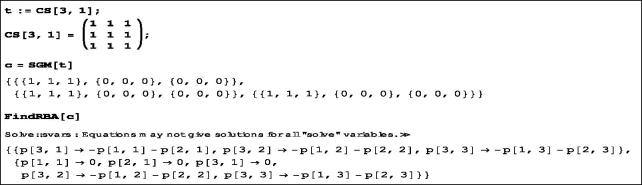
Results of the Mathematica code on *CS*(1).

We illustrate these functions by considering the first semigroup t=CS(1) of Table 1; for the detailed computation we refer to Section 4.2.1. The underlying set $\left\{e_{1},e_{2},e_{3}\right\}$ of *CS*(1) here will be simplified to {1, 2, 3}. The *Mathematica* code above yields the results given in Figure 2. In fact, the output gives two Rota-Baxter operators for *CS*(1), but the second is a special case of the first. Let p(1,1)=a,p(2,1)=b,p(1,2)=c,p(2,2)=d,p(1,3)=e,p(2,3)=f, where a,b,c,d,e,f∈k. Then p(3,1)=−a−b,p(3,2)=−c−d and p(3,3)=−e−f, so we obtain the matrix

$$\left(\begin{array}{ccc} a & c & e\\ b & d & f\\ -a-b & -c-d & -e-f \end{array}\right)\;(a,b,c,d,e,f\in k),$$

the transpose of which is given in Table 3 as $C_{1,1}$.

**Table 3.** RBOs on commutative semigroup algebras of order 3.

CS of order 3	Matrices of RBOs on semigroup algebras	CS of order 3	Matrices of RBOs on semigroup algebras
*CS*(1)	$C_{1,1}:=\left(\begin{array}{ccc} a & b & -a-b\\ c & d & -c-d\\ e & f & -e-f \end{array}\right)$	*CS*(2)	$\begin{array}{cc} & C_{2,1}:=\left(\begin{array}{ccc} a & -a & 0\\ b & -b & 0\\ c & -c & 0 \end{array}\right),\\ & C_{2,2}:=\left(\begin{array}{ccc} a & -a & 0\\ b & -b & 0\\ c & 2(b-a)-c & 2(a-b) \end{array}\right)(a\neq b) \end{array}$
*CS*(3)	$C_{3,1}:=\left(\begin{array}{ccc} a & 0 & -a\\ b & 0 & -b\\ c & 0 & -c \end{array}\right)$	*CS*(4)	$C_{4,1}:=\left(\begin{array}{ccc} 0 & 0 & 0\\ 0 & 0 & 0\\ 0 & 0 & 0 \end{array}\right)$
*CS*(5)	$C_{5,1}:=\left(\begin{array}{ccc} 0 & a & -a\\ 0 & b & -b\\ 0 & c & -c \end{array}\right)$	*CS*(6)	$C_{6,1}:=\left(\begin{array}{ccc} 0 & 0 & 0\\ 0 & 0 & 0\\ 0 & 0 & 0 \end{array}\right)$
*CS*(7)	$C_{7,1}:=\left(\begin{array}{ccc} a & 0 & -a\\ b & 0 & -b\\ a & 0 & -a \end{array}\right)$	*CS*(8)	$C_{8,1}:=\left(\begin{array}{ccc} 0 & 0 & 0\\ 0 & 0 & 0\\ 0 & 0 & 0 \end{array}\right)$
*CS*(9)	$C_{9,1}:=\left(\begin{array}{ccc} a & -a & 0\\ b & -b & 0\\ c & -c & 0 \end{array}\right)$	*CS*(10)	$C_{10,1}:=\left(\begin{array}{ccc} 0 & 0 & 0\\ 0 & 0 & 0\\ 0 & 0 & 0 \end{array}\right)$
*CS*(11)	$C_{11,1}:=\left(\begin{array}{ccc} 0 & a & -a\\ 0 & b & -b\\ 0 & c & -c \end{array}\right)$	*CS*(12)	$C_{12,1}:=\left(\begin{array}{ccc} 0 & 0 & 0\\ 0 & 0 & 0\\ 0 & 0 & 0 \end{array}\right)$

## 3. Rota-Baxter operators on semigroup algebras of order 2

In this section, we determine all Rota-Baxter operators on semigroup algebras k[S] of order 2.

As is well known [37], there are exactly five distinct nonisomorphic semigroups of order 2. We use $N_{2},L_{2},R_{2},Y_{2}$ and *Z*_2_ respectively to denote the null semigroup of order 2, the left zero semigroup, right zero semigroup, the semilattice of order 2 and the cyclic group of order 2. Since *L*_2_ and *R*_2_ are anti-isomorphic, there are exactly four distinct semigroups of order 2, up to isomorphism and anti-isomorphism, namely *N*_2_, *Y*_2_, *Z*_2_ and *L*_2_. Let $\left\{e_{1},e_{2}\right\}$ denote the underlying set of each semigroup. Then the Cayley tables for these semigroups are as follows:

Theorem 3.1. *Let* k *be a field of characteristics zero. All Rota-Baxter operators on a semigroup algebra* k[S] *of order 2 have their matrices C_P_ given in* *Table 4**, where all the parameters are in* k *and RBO* (*resp. SA*) *is the abbreviation of Rota-Baxter operator* (*resp. semigroup algebra*).

**Table 4.** Table of RBOs on two-dimensional semigroup algebras.

Semigroup of order 2	RBOs on SA	Semigroup of order 2	RBOs on SA
*N*_2_	$\left(\begin{array}{cc} a & -a\\ b & -b \end{array}\right)$	*Z*_2_	$\left(\begin{array}{cc} 0 & 0\\ 0 & 0 \end{array}\right)$
*Y*_2_	$\left(\begin{array}{cc} 0 & 0\\ 0 & 0 \end{array}\right)$	*L*_2_	$\left(\begin{array}{cc} a & -\frac{a^{2}}{b}\\ b & -a \end{array}\right)(b\neq 0),\left(\begin{array}{cc} 0 & a\\ 0 & 0 \end{array}\right)$

Proof. We divide the proof of the theorem into four cases, one for each of the four semigroups *S* in Table 5. For each case, by Theorem 2.1, *P* is a RBO on k[S] if and only if the eight Equations (6) hold (with 1⩽i,j,m⩽2). So we just need to solve these equations. It is straightforward to verify that what we obtain does satisfy all equations. Let $0_{2\times 2}$ denote the 2 × 2 zero matrix.

**Table 5.** The Cayley tables of semigroups of order 2.

$N_{2}:=\begin{array}{ccc} \cdot & e_{1} & e_{2}\\ e_{1} & e_{1} & e_{1}\\ e_{2} & e_{1} & e_{1} \end{array}$	$Y_{2}:=\begin{array}{ccc} \cdot & e_{1} & e_{2}\\ e_{1} & e_{1} & e_{1}\\ e_{2} & e_{1} & e_{2} \end{array}$	$Z_{2}:=\begin{array}{ccc} \cdot & e_{1} & e_{2}\\ e_{1} & e_{1} & e_{2}\\ e_{2} & e_{2} & e_{1} \end{array}$	$L_{2}:=\begin{array}{ccc} \cdot & e_{1} & e_{2}\\ e_{1} & e_{1} & e_{1}\\ e_{2} & e_{2} & e_{2} \end{array}$

**Case 1.** Let *S* = *N*_2_: In Eq. (6), taking i=j=1 with 1⩽m⩽2 and i=j=2 with 1⩽m⩽2, we get

(7) $${\left(c_{11}+c_{12}\right)}^{2}=2c_{11}(c_{11}+c_{12}),$$

(8) $$c_{12}(c_{11}+c_{12})=0,$$

(9) $${\left(c_{21}+c_{22}\right)}^{2}=2c_{11}(c_{21}+c_{22}),$$

(10) $$c_{12}(c_{21}+c_{22})=0.$$

Assume $c_{11}+c_{12}\neq 0$. Then by Eqs. (7) and (8), we have $c_{11}=c_{12}=0$, a contradiction to $c_{11}+c_{12}\neq 0$. Thus $c_{11}+c_{12}=0$. Assume $c_{21}+c_{22}\neq 0$. Then by Eq. (10), we get $c_{12}=0$, and so $c_{11}=0$. Then by Eq. (9), we have $c_{21}+c_{22}=0$, a contradiction. Thus $c_{21}+c_{22}=0$. Therefore Eqs. (7)–(10) are equivalent to

$$\{\begin{array}{c} c_{11}+c_{12}=0,\\ c_{21}+c_{22}=0. \end{array}$$

Denoting $a=c_{11}$ and $b=c_{21}$, we see that solutions ${\left(c_{ij}\right)}_{1⩽i,j⩽2}$ of Eqs. (7)–(10) are of the form

$$\left(\begin{array}{cc} a & -a\\ b & -b \end{array}\right)\;(a,b\in k).$$

It is straightforward to check that they also satisfy the other equations in Eq. (6). Hence these are all the matrices *C_P_* for Rota-Baxter operators on k[S].

**Case 2.** Let *S* = *Y*_2_: In Eq. (6), taking i=j=1 with 1⩽m⩽2 and i=j=2 with 1⩽m⩽2, we obtain

(11) $$c_{11}^{2}+2c_{11}c_{12}=2c_{11}(c_{11}+c_{12}),$$

(12) $$c_{12}^{2}=2c_{12}(c_{11}+c_{12}),$$

(13) $$c_{21}^{2}+2c_{21}c_{22}=2c_{21}(c_{11}+c_{22}),$$

(14) $$c_{22}^{2}=2(c_{12}c_{21}+c_{22}^{2}).$$

From Eq. (11) we have $c_{11}=0$. Then from Eq. (12) we have $c_{12}=0$. Thus Eq. (14) gives $c_{22}=0$. Further by Eq. (13), we have $c_{21}=0$. Thus the only solution is the zero solution $0_{2\times 2}$.

**Case 3.** *S* = *Z*_2_: In Eq. (6), taking i=j=1 with 1⩽m⩽2; i=1,j=2 with *m* = 2 and i=j=2 with *m* = 1, we obtain

(15) $$c_{11}^{2}+c_{12}^{2}=2c_{11}^{2}+2c_{12}c_{21},$$

(16) $$c_{11}c_{12}=c_{12}(c_{11}+c_{22}),$$

(17) $$c_{11}c_{22}+c_{12}c_{21}=c_{22}(c_{11}+c_{22})+c_{12}(c_{12}+c_{21}),$$

(18) $$c_{21}^{2}+c_{22}^{2}=2(c_{21}^{2}+c_{11}c_{22}).$$

By Eq. (16) we have $c_{12}c_{22}=0$. From Eq. (17) we get $c_{12}^{2}+c_{22}^{2}=0$. Thus $c_{12}=c_{22}=0$. Then Eqs. (15) and (18) give $c_{11}=c_{21}=0$. Thus the only solution is the zero solution $0_{2\times 2}.$

**Case 4.** *S* = *L*_2_: In Eq. (6), taking i=j=1 with 1⩽m⩽2 and i=2,j=1 with 1⩽m⩽2, we get

(19) $$c_{11}(c_{11}+c_{12})=c_{11}(2c_{11}+c_{12})+c_{12}c_{21},$$

(20) $$c_{12}(c_{11}+c_{12})=c_{12}(2c_{11}+c_{12}+c_{22}),$$

(21) $$c_{21}(c_{11}+c_{12})=c_{21}(2c_{11}+c_{12}+c_{22}),$$

(22) $$c_{22}(c_{11}+c_{12})=c_{21}c_{12}+c_{22}(c_{11}+c_{12}+c_{22}).$$

By Eqs. (19) and (22), we have $c_{11}^{2}=c_{22}^{2}$. By Eqs. (20) and (21), we get $c_{12}(c_{11}+c_{22})=0$ and $c_{21}(c_{11}+c_{22})=0$. Assume $c_{11}+c_{22}\neq 0$. Then $c_{12}=c_{21}=0$. So by Eq. (19), we have $c_{11}=0$ and then $c_{22}=0$, a contradiction. Thus $c_{11}+c_{22}=0$. Then Eqs. (19)–(22) are equivalent to the system of equations

$$\{\begin{array}{l} c_{11}+c_{22}=0,\\ c_{11}^{2}+c_{12}c_{21}=0. \end{array}$$

Denoting $a=c_{11}$ and $b=c_{21}$, then $c_{22}=-a$. When b≠0, then we also have $c_{12}=-\frac{a^{2}}{b}$. This gives the solutions

$$\left(\begin{array}{cc} a & -\frac{a^{2}}{b}\\ b & -a \end{array}\right)\;(b\neq 0,a\in k).$$

On the other hand, when *b* = 0, then $c_{11}=c_{22}=0$. Denoting $a=c_{12}$, we get the solutions

$$\left(\begin{array}{cc} 0 & a\\ 0 & 0 \end{array}\right)\;(a\in k).$$

These solutions to Eqs. (19)–(22) also satisfy the other equations in Eq. (6). Thus they give all the Rota-Baxter operators on k[S].

This completes the proof of Theorem 3.1. □

## 4. Rota-Baxter operators on commutative semigroup algebras of order 3

Up to isomorphism and anti-isomorphism, there are 18 semigroups of order 3 [12, 15, 18]. The Cayley tables of the 18 semigroups of order 3 can be found in [18]. See also [12, 29, 36]. We denote by *CS* and *NCS* the class of 12 commutative semigroups and the class of 6 noncommutative semigroups, respectively.

When a semigroup *S* has order 3, the equations in Eq. (6) for the matrix *C_P_* of a Rota-Baxter operator *P* on k[S] are given by the following 27 equations.

(23) $$\sum_{k=1}^{3}\sum_{\ell =1}^{3}r_{k\ell }^{m}c_{ik}c_{j\ell }=\sum_{k=1}^{3}\sum_{\ell =1}^{3}\left(r_{kj}^{\ell }c_{ik}+r_{ik}^{\ell }c_{jk}\right)c_{\ell m}\;(1⩽i,j,m⩽3).$$

In this section, we determine the Rota-Baxter operators on the semigroup algebras for the 12 commutative semigroups of order 3 in Table 1. Rota-Baxter operators on semigroup algebras for the 6 noncommutative semigroups of order 3 will be determined in Section 5.

### 4.1. Statement of the classification theorem in the commutative case

A classification of the 12 commutative semigroups of order 3 is given in Table 1.

We have the following classification of Rota-Baxter operators on order 3 commutative semigroup algebras. The proof will be given in Section 4.2.

Theorem 4.1. *Let* k *be a field of characteristic zero. The matrices of Rota-Baxter operators on 3-dimensional commutative semigroup algebras are given in* *Table 3**, where all the parameters take values in* k *and RBO* (*resp. CS*) *is the abbreviation for Rota-Baxter operator* (*resp. commutative semigroup*).

### 4.2. Proof of Theorem 4.1

We will prove Theorem 4.1 by considering each of the 12 commutative semigroups CS(i),1⩽i⩽12, of order 3 in Table 1. For each semigroup, we solve some of the equations in Eq. (23) for the Cayley table of the corresponding semigroup. It is straightforward to verify that what we obtain this way indeed satisfies all the equations in Eq. (23). Let $0_{3\times 3}$ denote the 3 × 3 zero matrix.

#### The proof for k[CS(1)]

4.2.1.

We prove that the matrices $C_{P}={\left(c_{ij}\right)}_{1⩽i,j⩽3}$ of all the Rota-Baxter operators *P* on the semigroup algebra k[CS(1)] are given by $C_{1,1}$ in Table 3.

Applying the Cayley table of *CS*(1) in Eq. (23) and taking i=j=1 with 1⩽m⩽3; *i* = 1,*j* = 2, 3 with *m* = 1 and *i* = 2, 3, *j* = 1 with *m* = 1, respectively, we obtain

(24) $${\left(c_{11}+c_{12}+c_{13}\right)}^{2}=2c_{11}(c_{11}+c_{12}+c_{13}),$$

(25) $$c_{12}(c_{11}+c_{12}+c_{13})=0,$$

(26) $$c_{13}(c_{11}+c_{12}+c_{13})=0,$$

(27) $$(c_{11}+c_{12}+c_{13})(c_{21}+c_{22}+c_{23})=c_{11}(c_{11}+c_{12}+c_{13}+c_{21}+c_{22}+c_{23}),$$

(28) $$(c_{11}+c_{12}+c_{13})(c_{31}+c_{32}+c_{33})=c_{11}(c_{11}+c_{12}+c_{13}+c_{31}+c_{32}+c_{33}),$$

(29) $${\left(c_{21}+c_{22}+c_{23}\right)}^{2}=2c_{11}(c_{21}+c_{22}+c_{23}),$$

(30) $${\left(c_{31}+c_{32}+c_{33}\right)}^{2}=2c_{11}(c_{31}+c_{32}+c_{33}).$$

Assume $c_{11}+c_{12}+c_{13}\neq 0.$ Then by Eqs. (24), (25) and (26), we get

$$2c_{11}=c_{11}+c_{12}+c_{13}\;\text{and}\;c_{12}=c_{13}=0.$$

So we have $c_{11}=c_{12}=c_{13}=0$. This contradicts $c_{11}+c_{12}+c_{13}\neq 0$. Thus $c_{11}+c_{12}+c_{13}=0$. By Eqs. (27) and (28), we have $c_{11}(c_{21}+c_{22}+c_{23})=0$ and $c_{11}(c_{31}+c_{32}+c_{33})=0.$ Then by Eqs. (29) and (30), we get ${\left(c_{21}+c_{22}+c_{23}\right)}^{2}=0$ and ${\left(c_{31}+c_{32}+c_{33}\right)}^{2}=0$. So we have $c_{21}+c_{22}+c_{23}=0$ and $c_{31}+c_{32}+c_{33}=0$. From these discussions, we see that Eqs. (24)–(30) are equivalent to

$$\{\begin{array}{c} c_{11}+c_{12}+c_{13}=0,\\ c_{21}+c_{22}+c_{23}=0,\\ c_{31}+c_{32}+c_{33}=0. \end{array}$$

So we have

$$\{\begin{array}{c} c_{13}=-c_{11}-c_{12},\\ c_{23}=-c_{21}-c_{22},\\ c_{33}=-c_{31}-c_{32}. \end{array}$$

Denote $a=c_{11},b=c_{12},c=c_{21},d=c_{22},e=c_{31},f=c_{32}$. Thus solutions ${\left(c_{ij}\right)}_{1⩽i,j⩽3}$ of Eqs. (24)–(30) are given by

$$C_{11}=\left(\begin{array}{ccc} a & b & -a-b\\ c & d & -c-d\\ e & f & -e-f \end{array}\right)\;(a,b,c,d,e,f\in k).$$

Since they can be checked to satisfy other equations in Eq. (23), they give the matrices of all the Rota-Baxter operators on k[CS(1)].

#### The proof for k[CS(2)]

4.2.2.

Here we prove that the matrices $C_{P}={\left(c_{ij}\right)}_{1⩽i,j⩽3}$ of all the Rota-Baxter operators *P* on the semigroup algebra k[CS(2)] are given by $C_{2,1}$ and $C_{2,2}$ in Table 3.

Applying the Cayley table of *CS*(2) in Eq. (23) and taking i=j=1 with 1⩽m⩽3 and 1⩽i⩽3,j=2 with *m* = 1, 2, we obtain

(31) $$(c_{11}+c_{12})(c_{11}+c_{12}+c_{13})+c_{13}(c_{11}+c_{12})=2c_{11}(c_{11}+c_{12}+c_{13}),$$

(32) $$c_{13}^{2}=2c_{12}(c_{11}+c_{12}+c_{13}),$$

(33) $$c_{13}(c_{11}+c_{12}+c_{13})=0,$$

(34) $$(c_{11}+c_{12})(c_{21}+c_{22}+c_{23})+c_{13}(c_{21}+c_{22})=c_{11}(c_{11}+c_{12}+c_{13}+c_{21}+c_{22}+c_{23}),$$

(35) $$c_{12}(c_{11}+c_{12}+c_{13}+c_{21}+c_{22}+c_{23})=0,$$

(36) $$(c_{21}+c_{22})(c_{21}+c_{22}+c_{23})+c_{23}(c_{21}+c_{22})=2c_{11}(c_{21}+c_{22}+c_{23}),$$

(37) $$c_{23}^{2}=2c_{12}(c_{21}+c_{22}+c_{23}),$$

(38) $$(c_{31}+c_{32})(c_{31}+c_{32}+c_{33})+c_{33}(c_{31}+c_{32})=2c_{11}(c_{31}+c_{32})+2c_{33}c_{21},$$

(39) $$c_{33}^{2}=2c_{12}(c_{31}+c_{32})+2c_{33}c_{22}.$$

Assume $c_{11}+c_{12}+c_{13}\neq 0$. Then by Eqs. (32) and (33), we have $c_{13}=c_{12}=0$. By Eq. (31), we have $c_{11}=0$. This contradicts $c_{11}+c_{12}+c_{13}\neq 0$. Thus we have $c_{11}+c_{12}+c_{13}=0$. By Eq. (32), we have $c_{13}=0$. So $c_{11}+c_{12}=0$. Then by Eqs. (34) and (35), we get $c_{11}(c_{21}+c_{22}+c_{23})=0$ and $c_{12}(c_{21}+c_{22}+c_{23})=0$. From Eq. (37), we obtain $c_{23}=0$. Then Eq. (36) gives $c_{21}+c_{22}=0$. Adding Eqs. (38) and (39), we get ${\left(c_{31}+c_{32}+c_{33}\right)}^{2}=0$. So $c_{31}+c_{32}+c_{33}=0$. By Eq. (39), we have $c_{33}^{2}=2c_{33}(c_{22}-c_{12})$. Thus $c_{33}=0$ or $c_{33}=2(c_{22}-c_{12}).$ Denote $a=c_{11},b=c_{21},c=c_{31}$. Then $c_{12}=-a$ and $c_{22}=-b$. We consider two cases.

**Case 1.** Suppose $c_{33}=0$: Then $c_{31}+c_{32}=0$. Then we have $c_{32}=-c$. Thus we get the solutions

$$C_{21}=\left(\begin{array}{ccc} a & -a & 0\\ b & -b & 0\\ c & -c & 0 \end{array}\right)\;(a,b\in k).$$

**Case 2.** Suppose $c_{33}\neq 0$: Then $c_{33}=2(c_{22}-c_{12})$. Thus

$$c_{33}=2(a-b)\;\text{and}\;c_{31}+c_{32}=-c_{33}=2(c_{12}-c_{22})=2(b-a).$$

So $c_{32}=2(b-a)-c$. Thus we get the solutions

$$C_{22}=\left(\begin{array}{ccc} a & -a & 0\\ b & -b & 0\\ c & 2(b-a)-c & 2(a-b) \end{array}\right)\;(a,b\in k,a\neq b).$$

They also satisfy the other equations in Eq. (23) hence give all the Rota-Baxter operators on k[CS(2)].

#### The proof for k[CS(3)]

4.2.3.

Here we prove that the matrices $C_{P}={\left(c_{ij}\right)}_{1⩽i,j⩽3}$ of all the Rota-Baxter operators *P* on the semigroup algebra k[CS(3)] are given by $C_{3,1}$ in Table 3.

Applying the Cayley table of *CS*(3) in Eq. (23) and taking i=1,j=1 with *m* = 2; i=1,j=2 with *m* = 1, 3; i=2,j=2 with 1⩽m⩽3 and i=3,j=3 with 1⩽m⩽3, we obtain

(40) $$c_{12}^{2}=2c_{12}(c_{11}+c_{12}+c_{13}),$$

(41) $$c_{13}(c_{21}+c_{22}+c_{23})+c_{12}c_{23}=c_{11}(c_{11}+c_{13}),$$

(42) $$c_{13}(c_{11}+c_{13}+c_{21}+c_{22}+c_{23})+c_{12}c_{23}=0,$$

(43) $$(c_{21}+c_{23})(c_{21}+2c_{22}+c_{23})=2(c_{11}(c_{21}+c_{23})+c_{22}c_{21}),$$

(44) $$c_{22}^{2}=2(c_{12}(c_{21}+c_{23})+c_{22}^{2}),$$

(45) $$c_{13}(c_{21}+c_{23})+c_{22}c_{23}=0,$$

(46) $$(c_{31}+c_{33})(c_{31}+c_{32}+c_{33})+c_{32}(c_{31}+c_{33})=2c_{11}(c_{31}+c_{32}+c_{33}),$$

(47) $$c_{32}^{2}=2c_{12}(c_{31}+c_{32}+c_{33}),$$

(48) $$c_{13}(c_{31}+c_{32}+c_{33})=0.$$

Eq. (42) gives

$$c_{13}(c_{21}+c_{22}+c_{23})+c_{12}c_{23}=-c_{13}(c_{11}+c_{13}).$$

Thus by Eq. (41), we have ${\left(c_{11}+c_{13}\right)}^{2}=0,$ and so $c_{11}+c_{13}=0$. Then by Eq. (40), we have $c_{12}=0$. Thus Eq. (44) gives $c_{22}=0$. By Eq. (45), we have $c_{13}(c_{21}+c_{23})=0$, and so $c_{11}(c_{21}+c_{23})=0$. Then Eq. (43) gives $c_{21}+c_{23}=0$. By Eq. (47), we have $c_{32}=0$. Then by Eqs. (46) and (48), we can obtain $c_{31}+c_{33}=0$. Let $a=c_{11},b=c_{21},c=c_{31}$. Thus solutions of Eqs. (40)–(48) are given by

$$C_{3,1}=\left(\begin{array}{ccc} a & 0 & -a\\ b & 0 & -b\\ c & 0 & -c \end{array}\right)\;(a,b,c\in k).$$

It can be checked that they also satisfy the other equations in Eq. (23) and hence give all the Rota-Baxter operators on k[CS(3)].

#### The proof for k[CS(4)]

4.2.4.

We prove that the matrices $C_{P}={\left(c_{ij}\right)}_{1⩽i,j⩽3}$ of all the Rota-Baxter operators *P* on the semigroup algebra k[CS(4)] are given by $C_{4,1}$ in Table 3.

Applying the Cayley table of *CS*(4) in Eq. (23) and taking i=j=1 with 1⩽m⩽3; i=j=2 with 1⩽m⩽3 and i=j=3 with 1⩽m⩽3, we obtain

(49) $$c_{11}^{2}=2c_{12}c_{13},$$

(50) $$c_{12}^{2}+2c_{12}(c_{11}+c_{13})=0,$$

(51) $$c_{13}^{2}+2c_{13}(c_{11}+c_{12})=0,$$

(52) $$c_{21}(c_{21}+c_{23})+c_{22}c_{23}+c_{23}(c_{21}+c_{22})=2c_{11}(c_{21}+c_{23}),$$

(53) $$c_{22}^{2}+2c_{12}(c_{21}+c_{23})=0,$$

(54) $$c_{23}^{2}=2c_{13}(c_{21}+c_{23})+2c_{22}c_{23},$$

(55) $$c_{31}(c_{31}+c_{32})+c_{32}(c_{31}+c_{32})+c_{32}c_{33}=2c_{11}(c_{31}+c_{32}),$$

(56) $$c_{32}^{2}=2c_{12}(c_{31}+c_{32})+2c_{33}c_{32},$$

(57) $$c_{33}^{2}+2c_{13}(c_{31}+c_{32})=0.$$

By Eqs. (50) and (51) and using Eq. (49), we have ${\left(c_{11}+c_{12}\right)}^{2}=0$ and ${\left(c_{11}+c_{13}\right)}^{2}=0$. So $c_{11}+c_{12}=0$ and $c_{11}+c_{13}=0$. Then Eq. (49) gives $c_{11}^{2}=2c_{11}^{2}$. Thus $c_{11}=0$. Then we get $c_{11}=c_{12}=c_{13}=0$. By Eq. (53), we have $c_{22}=0$. Thus Eqs. (52) and (54) give $c_{23}=0$ and $c_{21}=0$. Eq. (57) gives $c_{33}=0$ and so Eqs. (55) and (56) give $c_{31}=c_{32}=0$. Thus the system in Eq. (23) only has the zero solution $C_{4,1}=0_{3\times 3}.$

#### The proofs for k[CS(i)] when 5⩽i⩽11

4.2.5.

The proofs for the semigroups CS(6),CS(8), and *CS*(10) are similar to the proof for *CS*(4). So their proofs are omitted here but could be found in the on-line version [23]. Likewise the proofs for the semigroups CS(5),CS(7),CS(9), and *CS*(11) are similar to the proof for *CS*(3) and hence is left in [23].

#### The proof for k[CS(12)]

4.2.6.

We finally prove that the matrices $C_{P}={\left(c_{ij}\right)}_{1⩽i,j⩽3}$ of the Rota-Baxter operators *P* on the semigroup algebra k[CS(12)] are given by $C_{12,1}$ in Table 3.

Applying the Cayley table of *CS*(12) in Eq. (23) and taking i=j=1 with 1⩽m⩽3; i=1,j=2 with *m* = 2, 3; i=1,j=3 with *m* = 2, 3; i=j=2 with 1⩽m⩽3; i=2,j=3 with *m* = 2, 3 and i=j=3 with 1⩽m⩽3, we obtain

(58) $$2c_{12}c_{13}=c_{11}^{2}+2c_{12}c_{21}+2c_{13}c_{31},$$

(59) $$c_{13}^{2}=2c_{12}c_{22}+2c_{13}c_{32},$$

(60) $$c_{12}^{2}=2c_{12}c_{23}+2c_{13}c_{33},$$

(61) $$c_{13}c_{23}=c_{12}c_{13}+c_{22}^{2}+c_{32}(c_{12}+c_{23}),$$

(62) $$c_{12}c_{22}=c_{13}^{2}+c_{23}c_{22}+c_{33}(c_{12}+c_{23}),$$

(63) $$c_{13}c_{33}=c_{12}^{2}+c_{22}(c_{13}+c_{32})+c_{32}c_{33},$$

(64) $$c_{12}c_{32}=c_{12}c_{13}+c_{23}(c_{13}+c_{32})+c_{33}^{2},$$

(65) $$2c_{22}c_{23}=2c_{23}c_{11}+c_{21}^{2}+2c_{22}c_{31},$$

(66) $$c_{23}^{2}=2c_{23}c_{12}+2c_{22}c_{32},$$

(67) $$c_{22}^{2}=2c_{23}c_{13}+2c_{22}c_{33},$$

(68) $$c_{23}c_{33}=c_{12}(c_{22}+c_{33})+c_{22}c_{23}+c_{32}^{2},$$

(69) $$c_{22}c_{32}=c_{13}(c_{22}+c_{33})+c_{23}^{2}+c_{33}c_{32},$$

(70) $$2c_{32}c_{33}=2c_{11}c_{32}+2c_{33}c_{21}+c_{31}^{2},$$

(71) $$c_{33}^{2}=2c_{12}c_{32}+2c_{33}c_{22},$$

(72) $$c_{32}^{2}=2c_{13}c_{32}+2c_{33}c_{23}.$$

Denote $a=c_{13},b=c_{23}$ and $c=c_{33}$. By Eqs. (66) and (71), we get

$$\{\begin{array}{c} 2c_{12}c_{22}c_{32}=b^{2}c_{12}-2bc_{12}^{2},\\ 2c_{12}c_{22}c_{32}=c^{2}c_{22}-2cc_{22}^{2}. \end{array}$$

So

(73) $$2bc_{12}^{2}-b^{2}c_{12}=2cc_{22}^{2}-c^{2}c_{22}.$$

By Eqs. (60) and (67), we have

$$\{\begin{array}{c} 4abc=2bc_{12}^{2}-4b^{2}c_{12}^{2},\\ 4abc=2cc_{22}^{2}-4c^{2}c_{22}. \end{array}$$

Thus

$$\{\begin{array}{c} 2bc_{12}^{2}-b^{2}c_{12}=4abc+3b^{2}c_{12},\\ 2cc_{22}^{2}-c^{2}c_{22}=4abc+3c^{2}c_{22}. \end{array}$$

Using Eq. (73), we obtain $b^{2}c_{12}=c^{2}c_{22}$, and so $bc_{12}^{2}=cc_{22}^{2}$. Similarly, by Eqs. (59) and (66), and Eqs. (60) and (72), we get $b^{2}c_{12}=a^{2}c_{32}$ and $bc_{12}^{2}=ac_{32}^{2}$, respectively. This means that

(74) $$b^{2}c_{12}=c^{2}c_{22}=a^{2}c_{32}$$

and

(75) $$bc_{12}^{2}=cc_{22}^{2}=ac_{32}^{2}.$$

We consider two cases.

**Case 1.** abc = 0: There are three subcases to consider.

**Subcase 1.** a = b = c = 0: Then by Eqs. (60), (68) and (61), we have $c_{12}=0,c_{32}=0$ and $c_{22}=0$. By Eqs. (58), (65) and (70), we obtain $c_{11}=c_{21}=c_{31}=0$. Thus the only solution is given by the zero matrix $C_{12,1}:=0_{3\times 3}$.

**Subcase 2.** Two of *a*, *b*, *c* are 0: Without loss of generality, we may assume a=b=0 and c≠0. From Eq. (72), we obtain $c_{32}=0$. Then by Eq. (64), we have *c* = 0, a contradiction. Thus this case can not occur.

**Subcase 3.** One of *a*, *b*, *c* is 0: We may assume without loss of generality that *a* = 0, b≠0 and c≠0. By Eq. (59), we have $c_{12}c_{22}=0$. Then $c_{12}=0$ or $c_{22}=0$. If $c_{12}=0$, then by Eq. (62), we have $c_{33}+c_{22}=0$, and so $c_{22}=-c\neq 0$. By Eq. (67), $3c^{2}=0$. Thus *c* = 0. This contradicts c≠0. If $c_{22}=0$, then by Eq. (62), $b=-c_{12}$. By Eq. (60), we have $3b^{2}=0$. Thus *b* = 0, a contradiction. We see that this case also can not occur.

**Case 2.** abc ≠0**:** Then a≠0,b≠0 and c≠0. If one of $c_{12},c_{22},c_{32}$ is 0, we may assume $c_{12}=0$, and then by Eqs. (74 and (75), we obtain $c_{22}=c_{32}=0$. So Eq. (59) gives $a^{2}=0$, a contradiction. Thus *c*_12_, *c*_22_ and *c*_32_ are nonzero. Since $b^{2}c_{12}=c^{2}c_{22}$ and $bc_{12}^{2}=cc_{22}^{2}$, we have $b^{2}c_{12}^{2}=bcc_{22}^{2}.$ So $c^{2}c_{22}c_{12}=bcc_{22}^{2}$. Thus $cc_{12}=bc_{22}$. Similarly, we have $ac_{12}=bc_{32}$ and $ac_{22}=cc_{32}.$ So $b^{3}c_{12}=c^{2}bc_{22}=c^{3}c_{12}$. Thus $a^{3}=c^{3}$. Similarly, we have $a^{3}=b^{3}$. By Eqs. (66) and (69), we get

$$b^{2}+2bc_{12}+2ac_{22}+2cc_{32}+2ac=0.$$

Since $ac_{22}=cc_{32}$, we have $b^{2}+2bc_{12}+4ac_{22}+2ac=0$. So $b^{3}+2b^{2}c_{12}+4abc_{22}+2abc=0$. Since $b^{2}c_{12}=c^{2}c_{22}$, we have

(76) $$2(c^{2}+2ab)c_{22}=-b^{3}-2abc=-c^{3}-2abc.$$

We further divide into two subcases.

**Subcase 1.** $c^{2}+2ab\neq 0$: By Eq. (76), we have $c_{22}=-\frac{c}{2}$. From $cc_{12}=bc_{22}$ and $ac_{22}=cr_{32}$, we obtain $c_{12}=-\frac{b}{2}$ and $c_{32}=-\frac{a}{2}$. By Eq. (59), we have $a^{2}=\frac{bc}{2}-a^{2}$. So $a^{2}=\frac{bc}{4}$. By Eq. (72), we get $\frac{5}{4}a^{2}=2bc$. Thus we get $a^{2}=\frac{8}{5}bc$. So by $a^{2}=\frac{bc}{4}$, we get *bc* = 0, a contradiction.

**Subcase 2.** $c^{2}+2ab=0$: Then $c^{4}=4a^{2}b^{2}$. Adding Eqs. (67) and (71), we obtain $c_{22}^{2}+2c_{12}c_{32}=0$. So we have $a^{2}b^{2}c_{22}^{2}+2a^{2}b^{2}c_{12}c_{32}=0$. By Eq. (74), we have $(a^{2}b^{2}+2c^{4})c_{22}^{2}=0$. Since $c_{22}\neq 0$, we have $a^{2}b^{2}+2c^{4}=0$. Then by $c^{4}=4a^{2}b^{2}$, we have $9{\left(ab\right)}^{2}=0$, and so *ab* = 0, again a contradiction.

In summary, the only solution for k[CS(12)] is the zero solution $0_{3\times 3}$, as claimed.

Now the proof of Theorem 4.1 is completed.

## 5. Rota-Baxter operators on noncommutative semigroup algebras of order 3

In this Section, we classify all Rota-Baxter operators on noncommutative semigroup algebras of order 3.

### 5.1. Statement of the classification theorem in the noncommutative case

A classification of the six noncommutative semigroups of order 3, up to isomorphism and anti-isomorphism, is given in Table 2.

For Rota-Baxter operators on the corresponding semigroup algebras, we have the following classification theorem whose proof will be given in Section 5.2

Theorem 5.1. *Let* k *be a field of characteristic zero that is closed under taking square root. The matrices of the Rota-Baxter operators on noncommutative semigroup algebras of order three are given in Table 6, where all the parameters take values in* k *and i denotes* $\sqrt{-1}$ *as usual.*

**Table 6.** RBOs on noncomumutative semigroup algebras of order 3.

Semigroups	Matrices of Rota-Baxter operators on semigroup algebras
NCS(1)	$\begin{array}{l} N_{1,1}=\left(\begin{array}{ccc} 0 & 0 & 0\\ 0 & 0 & 0\\ 0 & a & 0 \end{array}\right),N_{1,2}=\left(\begin{array}{ccc} 0 & 0 & 0\\ 0 & 0 & 0\\ -a & a & 0 \end{array}\right)(a\neq 0),N_{1,3}=\left(\begin{array}{ccc} 0 & 0 & 0\\ -b+c & -c & b\\ \frac{c^{2}-bc}{b} & -\frac{c^{2}}{b} & c \end{array}\right)(a\neq 0,b\neq 0),\\ N_{1,4}=\left(\begin{array}{ccc} 0 & 0 & 0\\ -a & -b & a\\ -b & -\frac{b^{2}}{a} & b \end{array}\right)(a\neq 0),N_{1,5}=\left(\begin{array}{ccc} a & 0 & -a\\ -b & 0 & b\\ a & 0 & -a \end{array}\right)(a\neq 0) \end{array}$
NCS(2)	$\begin{array}{l} N_{2,1}=\left(\begin{array}{ccc} 0 & 0 & 0\\ c & 0 & -c\\ 0 & 0 & 0 \end{array}\right)(a\neq 0),N_{2,2}=\left(\begin{array}{ccc} 0 & 0 & 0\\ 0 & 0 & 0\\ a & 0 & 0 \end{array}\right),N_{2,3}=\left(\begin{array}{ccc} 0 & 0 & 0\\ 0 & 0 & 0\\ 0 & b & 0 \end{array}\right),\\ N_{2,4}=\left(\begin{array}{ccc} b & 0 & 0\\ b & 0 & 0\\ -\frac{b^{2}}{a} & 0 & -b \end{array}\right)(a\neq 0,b\neq 0,a+b\neq 0),N_{2,5}=\left(\begin{array}{ccc} 0 & 0 & a\\ 0 & 0 & 0\\ 0 & 0 & 0 \end{array}\right)(a\neq 0),\\ N_{2,6}=\left(\begin{array}{ccc} 0 & 0 & a\\ 0 & 0 & a\\ 0 & 0 & 0 \end{array}\right)(a\neq 0),N_{2,7}=\left(\begin{array}{ccc} -a & 0 & a\\ c & 0 & -c\\ -a & 0 & a \end{array}\right)(a\neq 0),\\ N_{2,8}=\left(\begin{array}{ccc} -a & b & a\\ -a & b & a\\ b-a & \frac{b(a-b)}{a} & a-b \end{array}\right)(a\neq 0,b\neq 0),N_{2,9}=\left(\begin{array}{ccc} 0 & b & 0\\ 0 & 0 & 0\\ 0 & 0 & 0 \end{array}\right)(b\neq 0) \end{array}$
NCS(3)	$\begin{array}{l} N_{3,1}=\left(\begin{array}{ccc} 0 & a & -a\\ 0 & b & -b\\ 0 & b & -b \end{array}\right)(a\neq 0),N_{3,2}=\left(\begin{array}{ccc} 0 & 0 & 0\\ 0 & -\sqrt{ab}i & b\\ 0 & a & \sqrt{ab}i \end{array}\right)(a\neq 0,b\neq 0),\\ N_{3,3}=\left(\begin{array}{ccc} 0 & 0 & 0\\ 0 & \sqrt{ab}i & b\\ 0 & a & -\sqrt{ab}i \end{array}\right)(a\neq 0,b\neq 0),N_{3,4}=\left(\begin{array}{ccc} 0 & 0 & 0\\ 0 & 0 & 0\\ 0 & a & 0 \end{array}\right)(a\neq 0),\\ N_{3,5}=\left(\begin{array}{ccc} 0 & 0 & 0\\ 0 & 0 & 0\\ -a & a & 0 \end{array}\right)(a\neq 0),N_{3,6}=\left(\begin{array}{ccc} 0 & 0 & 0\\ c & -(b+c) & b\\ \frac{c(b+c)}{b} & -\frac{{\left(b+c\right)}^{2}}{b} & b+c \end{array}\right)(a\neq 0,b\neq 0,b+c\neq 0),\\ N_{3,7}=\left(\begin{array}{ccc} 0 & 0 & 0\\ 0 & 0 & b\\ 0 & 0 & 0 \end{array}\right),N_{3,8}=\left(\begin{array}{ccc} 0 & 0 & 0\\ -b & 0 & b\\ 0 & 0 & 0 \end{array}\right)(b\neq 0) \end{array}$
NCS(4)	$\begin{array}{l} N_{4,1}=\left(\begin{array}{ccc} -a & 0 & a\\ b & 0 & -b\\ c & 0 & -c \end{array}\right)(a\neq 0,a+b\neq 0),N_{4,2}=\left(\begin{array}{ccc} -a & 0 & a\\ -a & 0 & a\\ c & d & -c-d \end{array}\right)(a\neq 0),\\ N_{4,3}=\left(\begin{array}{ccc} 0 & 0 & 0\\ -a & 0 & a\\ b & 0 & -b \end{array}\right)(a\neq 0),N_{4,4}=\left(\begin{array}{ccc} 0 & 0 & 0\\ 0 & 0 & 0\\ b & c & -(b+c) \end{array}\right)(c\neq 0),\\ N_{4,5}=\left(\begin{array}{ccc} 0 & 0 & 0\\ a & 0 & 0\\ b & 0 & -b \end{array}\right),N_{4,6}=\left(\begin{array}{ccc} a & -(a+b) & b\\ a & -(a+b) & b\\ c & d & -(c+d) \end{array}\right)(b\neq 0,a+b\neq 0),\\ N_{4,7}=\left(\begin{array}{ccc} a & b & 0\\ -\frac{a^{2}}{b} & -a & 0\\ c & b & a-c \end{array}\right)(a+b\neq 0,b\neq 0),N_{4,8}=\left(\begin{array}{ccc} -b & b & 0\\ -b & b & 0\\ c & d & -c-d \end{array}\right)(b\neq 0) \end{array}$
NCS(5)	$\begin{array}{l} N_{5,1}=\left(\begin{array}{ccc} \frac{ac}{F} & \frac{ab}{F} & a\\ \frac{cd}{F} & \frac{bd}{F} & d\\ c & b & F \end{array}\right),N_{5,2}=\left(\begin{array}{ccc} -\frac{ac}{F} & -\frac{ab}{F} & a\\ -\frac{cd}{F} & -\frac{bd}{F} & d\\ c & b & -F \end{array}\right)(a,b,c,d\in k\setminus \{0\}),F:=\sqrt{-(ac+bd)},\\ N_{5,3}=\left(\begin{array}{ccc} 0 & 0 & 0\\ -\frac{c\sqrt{d}}{\sqrt{b}}i & -\sqrt{bd}i & d\\ c & b & \sqrt{bd}i \end{array}\right),N_{5,4}=\left(\begin{array}{ccc} 0 & 0 & 0\\ \frac{c\sqrt{d}}{\sqrt{b}}i & \sqrt{bd}i & d\\ c & b & -\sqrt{bd}i \end{array}\right)(b,c,d\in k\setminus \{0\}),\\ N_{5,5}=\left(\begin{array}{ccc} -\sqrt{ac}i & 0 & a\\ -\frac{\sqrt{c}d}{\sqrt{a}}i & 0 & d\\ c & 0 & \sqrt{ac}i \end{array}\right),N_{5,6}=\left(\begin{array}{ccc} \sqrt{ac}i & 0 & a\\ \frac{\sqrt{c}d}{\sqrt{a}}i & 0 & d\\ c & 0 & -\sqrt{ac}i \end{array}\right)(a,c,d\in k\setminus \{0\}),\\ N_{5,7}=\left(\begin{array}{ccc} 0 & -\frac{a\sqrt{b}i}{\sqrt{d}} & a\\ 0 & -\sqrt{bd}i & d\\ 0 & b & \sqrt{bd}i \end{array}\right),N_{5,8}=\left(\begin{array}{ccc} 0 & \frac{a\sqrt{b}i}{\sqrt{d}} & a\\ 0 & \sqrt{bd}i & d\\ 0 & b & -\sqrt{bd}i \end{array}\right)(a,b,c,d\in k\setminus \{0\}),\\ N_{5,9}=\left(\begin{array}{ccc} -\sqrt{ac}i & -\frac{\sqrt{a}bi}{\sqrt{c}} & a\\ 0 & 0 & 0\\ c & b & \sqrt{ac}i \end{array}\right),N_{5,10}=\left(\begin{array}{ccc} \sqrt{ac}i & \frac{\sqrt{a}bi}{\sqrt{c}} & a\\ 0 & 0 & 0\\ c & b & -\sqrt{ac}i \end{array}\right)(a,b,c\in k\setminus \{0\}),\\ N_{5,11}=\left(\begin{array}{ccc} 0 & 0 & 0\\ 0 & -\sqrt{db}i & d\\ 0 & b & \sqrt{db}i \end{array}\right),N_{5,12}=\left(\begin{array}{ccc} 0 & 0 & 0\\ 0 & \sqrt{db}i & d\\ 0 & b & -\sqrt{db}i \end{array}\right)(b\neq 0,d\neq 0),\\ N_{5,13}=\left(\begin{array}{ccc} -\frac{eb}{c} & -\frac{eb^{2}}{c^{2}} & 0\\ e & \frac{eb}{c} & 0\\ c & b & 0 \end{array}\right)(b\neq 0,c\neq 0),N_{5,14}=\left(\begin{array}{ccc} \frac{ae}{d} & -\frac{a^{2}e}{d^{2}} & a\\ e & -\frac{ae}{d} & d\\ 0 & 0 & 0 \end{array}\right)(a\neq 0,d\neq 0),\\ N_{5,15}=\left(\begin{array}{ccc} -\sqrt{ac}i & 0 & a\\ 0 & 0 & 0\\ c & 0 & \sqrt{ac}i \end{array}\right),N_{5,16}=\left(\begin{array}{ccc} \sqrt{ac}i & 0 & a\\ 0 & 0 & 0\\ c & 0 & -\sqrt{ac}i \end{array}\right)(a\neq 0,c\neq 0),\\ N_{5,17}=\left(\begin{array}{ccc} 0 & 0 & 0\\ e & 0 & d\\ 0 & 0 & 0 \end{array}\right)(d\neq 0),N_{5,18}=\left(\begin{array}{ccc} 0 & 0 & 0\\ e & 0 & 0\\ c & 0 & 0 \end{array}\right)(c\neq 0),N_{5,19}=\left(\begin{array}{ccc} 0 & e & 0\\ 0 & 0 & 0\\ 0 & b & 0 \end{array}\right)(b\neq 0),\\ N_{5,20}=\left(\begin{array}{ccc} 0 & e & a\\ 0 & 0 & 0\\ 0 & 0 & 0 \end{array}\right)(a\neq 0),N_{5,21}=\left(\begin{array}{ccc} -\sqrt{ef}i & e & 0\\ f & \sqrt{ef}i & 0\\ 0 & 0 & 0 \end{array}\right),N_{5,22}=\left(\begin{array}{ccc} \sqrt{ef}i & e & 0\\ f & -\sqrt{ef}i & 0\\ 0 & 0 & 0 \end{array}\right) \end{array}$
NCS(6)	$\begin{array}{l} N_{6,1}=\left(\begin{array}{ccc} a & 0 & a\\ 0 & 0 & 0\\ -a & 0 & -a \end{array}\right)(a\neq 0),N_{6,2}=\left(\begin{array}{ccc} a & 0 & a\\ -c & 0 & c\\ -a & 0 & -a \end{array}\right)(a\neq 0),\\ N_{6,3}=\left(\begin{array}{ccc} \sqrt{ab}i & 0 & a\\ 0 & 0 & 0\\ b & 0 & -\sqrt{ab}i \end{array}\right)(a\neq 0,b\neq 0),N_{6,4}=\left(\begin{array}{ccc} -\sqrt{ab}i & 0 & a\\ 0 & 0 & 0\\ b & 0 & \sqrt{ab}i \end{array}\right)(a\neq 0,b\neq 0),\\ N_{6,5}=\left(\begin{array}{ccc} 0 & 0 & a\\ 0 & 0 & 0\\ 0 & 0 & 0 \end{array}\right)(a\neq 0),N_{6,6}=\left(\begin{array}{ccc} 0 & 0 & 0\\ a & 0 & -a\\ 0 & 0 & 0 \end{array}\right),N_{6,7}=\left(\begin{array}{ccc} 0 & 0 & 0\\ 0 & 0 & 0\\ a & 0 & 0 \end{array}\right)(a\neq 0),\\ N_{6,8}=\left(\begin{array}{ccc} 0 & 0 & 0\\ 0 & 0 & 0\\ a & -a & 0 \end{array}\right)(a\neq 0),N_{6,9}=\left(\begin{array}{ccc} \frac{ab}{a-b} & 0 & \frac{a^{2}}{b-a}\\ 0 & 0 & 0\\ \frac{b^{2}}{a-b} & b & \frac{ab}{b-a} \end{array}\right)(a\neq 0) \end{array}$

### 5.2. Proof of Theorem 5.1

We will prove Theorem 5.1 case by case for the six semigroups in Table 2.

#### The proof for k[NCS(1)]

5.2.1.

We first prove that the matrices ${\left(c_{ij}\right)}_{1⩽i,j⩽3}$ of the Rota-Baxter operators *P* on the semigroup algebra k[NCS(1)] are given by $N_{1,i},1⩽i⩽5$, in Table 6.

Applying the Cayley table of *NCS*(1) in Eq. (23) and then taking i=1,j=1,2 with 1⩽m⩽3; i=j=2 with 1⩽m⩽3; i=2,j=3 with *m* = 1; i=3,j=2 with 1⩽m⩽3 and i=j=3 with *m* = 1, 3, we obtain

(77) $$c_{11}^{2}=c_{13}^{2}+c_{12}c_{13},$$

(78) $$c_{12}^{2}=2c_{12}(c_{11}+c_{12}+c_{13}),$$

(79) $$2c_{13}^{2}+c_{13}(2c_{11}+c_{12})=0,$$

(80) $$c_{12}c_{23}+c_{13}(c_{21}+c_{23})=c_{11}^{2}+c_{13}c_{31},$$

(81) $$c_{12}(c_{11}+c_{21}+c_{22}+c_{23})+c_{13}c_{32}=0,$$

(82) $$c_{13}(c_{11}+c_{21}+c_{23})+c_{12}c_{23}+c_{13}c_{33}=0,$$

(83) $$c_{21}(c_{21}+c_{23})+c_{23}(c_{21}+c_{22}+c_{23})=c_{11}(2c_{21}+c_{23})+c_{23}c_{31},$$

(84) $$c_{22}^{2}+c_{23}c_{32}+c_{12}(2c_{21}+c_{23})=0,$$

(85) $$c_{23}(c_{22}+c_{33})+c_{13}(2c_{21}+c_{23})=0,$$

(86) $$(c_{21}+c_{22}+c_{23})(c_{31}+c_{33})=c_{11}(c_{21}+c_{22}+c_{23}+c_{31}+c_{33}),$$

(87) $$(c_{31}+c_{32}+c_{33})(c_{21}+c_{23})=c_{11}(c_{31}+c_{21}+c_{23})+c_{32}c_{21}+c_{31}c_{33},$$

(88) $$c_{12}(c_{31}+c_{21}+c_{23})+c_{32}(c_{33}+c_{22})=0,$$

(89) $$c_{33}^{2}+c_{23}c_{32}+c_{13}(c_{31}+c_{21}+c_{23})=0,$$

(90) $$(c_{31}+c_{33})(c_{31}+c_{32}+c_{33})=c_{11}(2c_{31}+c_{32}+2c_{33}),$$

(91) $$c_{13}(2c_{31}+c_{32}+2c_{33})=0.$$

By Eq. (79) we have $c_{12}c_{13}=-2c_{13}^{2}-2c_{11}c_{13}.$ Then by Eq. (77) we get ${\left(c_{11}+c_{13}\right)}^{2}=0.$ So we get $c_{11}+c_{13}=0$. Then Eq. (78) gives $c_{12}=0$. We divide the rest of the proof into two cases depending on whether or not $c_{11}=0$.

**Case 1.** $c_{11}=0$: Then $c_{13}=0$. There are two subcases to consider.

**Subcases 1.** $c_{23}=0$: Then by Eqs. (84) and (89), we have $c_{22}=c_{33}=0$. So by Eq. (83), we get $c_{21}=0$. Denote $a=c_{32}$, where a∈k. If $c_{31}=0$, then we get the solution

$$N_{1,1}=\left(\begin{array}{ccc} 0 & 0 & 0\\ 0 & 0 & 0\\ 0 & a & 0 \end{array}\right)\;(a\in k).$$

If $c_{31}\neq 0$, then by Eq. (90), we have $c_{31}+c_{32}=0$. Thus we get $c_{31}=-c_{32}=-a$. So a≠0. Thus we obtain the solution

$$N_{1,2}=\left(\begin{array}{ccc} 0 & 0 & 0\\ 0 & 0 & 0\\ -a & a & 0 \end{array}\right)\;(a\in k,a\neq 0).$$

**Subcase 2.** $c_{23}\neq 0$: Denote $a=c_{23}$. Then a≠0. By Eq. (85), we have $c_{22}+c_{33}=0$. Denote $b=c_{33}$. Then $c_{22}=-b$. Thus Eq. (84) gives $c_{32}=-\frac{b^{2}}{a}$. We subdivide further into two cases.

(1) If $c_{31}+c_{33}\neq 0$, then by Eq. (90), we have $c_{31}+c_{32}+c_{33}=0$. So we get

$$c_{31}=-c_{32}-c_{33}=\frac{b^{2}-ba}{a}.$$

Note that if $c_{33}=b=0$, then $c_{31}=0$, a contradiction. Thus b≠0, and so $c_{32}\neq 0$. By Eq. (86), we have $c_{21}+c_{22}+c_{23}=0$. Thus $c_{21}=b-a$. Thus we get the solutions

$$N_{1,3}=\left(\begin{array}{ccc} 0 & 0 & 0\\ b-a & -b & a\\ \frac{b(b-a)}{a} & -\frac{b^{2}}{a} & b \end{array}\right)\;(a,b\in k\setminus \{0\}).$$

(2) If $c_{31}+c_{33}=0,$ then $c_{31}=-b$. Since $c_{22}=-b,c_{31}-c_{22}=0$. Then by Eq. (83), we have ${\left(c_{21}+c_{23}\right)}^{2}=c_{23}(c_{31}-c_{22})=0$. Thus $c_{21}=-a$. Thus we obtain the solutions

$$N_{1,4}=\left(\begin{array}{ccc} 0 & 0 & 0\\ -a & -b & a\\ -b & -\frac{b^{2}}{a} & b \end{array}\right)\;(a,b\in k,a\neq 0).$$

**Case 2.** $c_{11}\neq 0$: Denote $a=c_{11}$. Then $c_{13}=-a\neq 0$. Since $c_{12}=0$, Eq. (81) becomes $c_{13}c_{32}=0$. Then $c_{32}=0$. So by Eq. (84), we obtain $c_{22}=0$. Further, by Eq. (91), we have $c_{31}+c_{33}=0$. Thus by Eq. (86), $c_{21}+c_{23}=0$.

We divide into two subcases to consider.

**Subcase 1.** $c_{23}=0$: Then $c_{21}=0$. By Eq. (80), $c_{11}^{2}+c_{13}c_{31}=0$. Then we obtain $c_{31}=a$. So we have $c_{33}=-a$. Thus we obtain the solutions

$$N_{1,5_{1}}:=\left(\begin{array}{ccc} a & 0 & -a\\ 0 & 0 & 0\\ a & 0 & -a \end{array}\right)\;(a\in k,a\neq 0).$$

**Subcase 2.** $c_{23}\neq 0$: Denote $b=c_{23}$. Then b≠0. Thus by $c_{21}+c_{23}=0$, we have $c_{21}=-b$ and by Eq. (85), $c_{13}c_{21}+c_{23}c_{33}=0$, we get $c_{33}=-a$. Then we solutions

$$N_{1,5_{2}}:=\left(\begin{array}{ccc} a & 0 & -a\\ -b & 0 & b\\ a & 0 & -a \end{array}\right)\;(a,b\in k\setminus \{0\}).$$

In summary, we get get solutions of Eqs. (77)–(91)

$$N_{1,5}=\left(\begin{array}{ccc} a & 0 & -a\\ -b & 0 & b\\ a & 0 & -a \end{array}\right)\;(a,b\in k,a\neq 0).$$

It can be checked that they also satisfy the other equations in Eq. (23) and hence give matrices of Rota-Baxter operators on k[NCS(1)].

#### The proof for k[NCS(i)] where i=2,3,4,6

5.2.2.

The proof of these cases are similar to the one for k[NCS(1)] in that the proofs are carried out by (iterated) bisecting depending on whether or not certain elements are zero. Details of the proofs are provided in [23]. So we next move on to the proof of k[NCS(5)].

#### The proof for k[NCS(5)]

5.2.3.

We next prove that the matrices of the Rota-Baxter operators on the semigroup algebra k[NCS(5)] are given by $N_{5,i},1⩽i⩽22,$ in Table 6.

Applying the Cayley table of *NCS*(5) in Eq. (23) and then taking i=j=1 with 1⩽m⩽3; i=2,j=1 with 1⩽m⩽3 and i=3,j=1 with 1⩽m⩽3, we obtain

(92) $$c_{11}^{2}+c_{12}c_{21}+c_{13}c_{31}=0,$$

(93) $$c_{11}c_{12}+c_{12}c_{22}+c_{13}c_{32}=0,$$

(94) $$c_{11}c_{13}+c_{12}c_{23}+c_{13}c_{33}=0,$$

(95) $$c_{21}c_{11}+c_{21}c_{22}+c_{23}c_{31}=0,$$

(96) $$c_{21}c_{12}+c_{22}^{2}+c_{23}c_{32}=0,$$

(97) $$c_{21}c_{13}+c_{22}c_{23}+c_{23}c_{33}=0,$$

(98) $$c_{11}c_{31}+c_{21}c_{32}+c_{31}c_{33}=0,$$

(99) $$c_{12}c_{31}+c_{22}c_{32}+c_{32}c_{33}=0,$$

(100) $$c_{13}c_{31}+c_{23}c_{32}+c_{33}^{2}=0.$$

From Eq. (100), we have $c_{33}^{2}=-(c_{13}c_{31}+c_{23}c_{32})$. Denote $a=c_{13},b=c_{32},c=c_{31}$, and $d=c_{23}$. Then $c_{33}^{2}=-ac-bd$. So we have $c_{33}=\pm \sqrt{-ac-bd}$. Denote $F:=\sqrt{-ac-bd}$. So $F^{2}=-ac-bd$ and $c_{33}=\pm F$.

We divide the proof into five cases.

**Case 1.** *a*, *b*, *c,* and *d* are nonzero: Then $c_{13},c_{32},c_{31}$ and *c*_23_ are nonzero. By Eqs. (94) and (98), we have

$$c_{31}c_{23}c_{12}=-c_{31}(c_{11}c_{13}+c_{13}c_{33})=-c_{13}(c_{11}c_{31}+c_{31}c_{33})=c_{13}c_{32}c_{21}.$$

Thus

(101) $$c_{21}=\frac{cd}{ab}c_{12}.$$

Then by Eqs. (98) and (101), we obtain

(102) $$c_{11}=-c_{33}-\frac{c_{32}}{c_{31}}c_{21}=-c_{33}-\frac{b}{c}c_{21}=-c_{33}-\frac{d}{a}c_{12}.$$

Applying Eqs. (97) and (101), we have

(103) $$c_{22}=-c_{33}-\frac{c_{13}}{c_{23}}c_{21}=-c_{33}-\frac{a}{d}c_{21}=-c_{33}-\frac{c}{b}c_{12}.$$

In Eq. (93), by replacing *c*_11_ and *c*_22_ by $-c_{33}-\frac{d}{a}c_{12}$ and $-c_{33}-\frac{c}{b}c_{12}$, respectively, we get

(104) $$c_{33}^{2}c_{12}^{2}-2abc_{33}c_{12}+{\left(ab\right)}^{2}=0.$$

If ac+bd=0, then $c_{33}=0$. Thus by Eq. (104), *ab* = 0, a contradiction. Thus we have ac+bd≠0. So $c_{33}\neq 0$ and F≠0. By Eq. (104) again, we get ${\left(c_{33}c_{12}-ab\right)}^{2}=0.$ Thus $c_{12}=\frac{ab}{c_{33}}$. Since $c_{33}=\pm F,c_{12}=\pm \frac{ab}{F}$. Then by Eq. (101), $c_{21}=\pm \frac{cd}{F}$. By Eqs. (102) and (103) and $F^{2}=-ac-bd$, we get $c_{11}=\pm \frac{ac}{F}$ and $c_{22}=\pm \frac{bd}{F}$. Thus we obtain the solutions

$$N_{5,1}=\left(\begin{array}{ccc} \frac{ac}{F} & \frac{ab}{F} & a\\ \frac{cd}{F} & \frac{bd}{F} & d\\ c & b & F \end{array}\right)\;\text{and}\;N_{5,2}=\left(\begin{array}{ccc} -\frac{ac}{F} & -\frac{ab}{F} & a\\ -\frac{cd}{F} & -\frac{bd}{F} & d\\ c & b & -F \end{array}\right)\;(a,b,c,d\in k\setminus \{0\}).$$

**Case 2.** Exactly one of a,b,c,d is 0: Then there are four subcases to consider.

**Subcase 1.** *a* = 0 and b,c,d≠0: Then $c_{13}=0,c_{32}\neq 0,c_{31}\neq 0$, and $c_{23}\neq 0$. By Eq. (94), we have $c_{12}c_{23}=0$. So $c_{12}=0$. Thus by Eq. (92), $c_{11}=0$. Then Eq. (97) gives $c_{22}+c_{33}=0$ and Eq. (100) gives $c_{33}=\pm \sqrt{bd}i$. So $c_{22}=\mp \sqrt{bd}i$. By Eq. (95), we have $c_{21}=-\frac{c_{23}c_{31}}{c_{22}}$. So $c_{21}=\mp \frac{c\sqrt{d}i}{\sqrt{b}}$. Thus we obtain the solutions

$$N_{5,3}=\left(\begin{array}{ccc} 0 & 0 & 0\\ -\frac{c\sqrt{d}i}{\sqrt{b}} & -\sqrt{bd}i & d\\ c & b & \sqrt{bd}i \end{array}\right)\;\text{and}\;N_{5,4}=\left(\begin{array}{ccc} 0 & 0 & 0\\ \frac{c\sqrt{d}i}{\sqrt{b}} & \sqrt{bd}i & d\\ c & b & -\sqrt{bd}i \end{array}\right)\;(a,b,c,d\in k,b,c,d\neq 0).$$

**Subcase 2.** *b* = 0 and a,c,d≠0: Then $c_{32}=0,c_{13}\neq 0,c_{31}\neq 0$ and $c_{23}\neq 0$. Then by Eq. (99), $c_{12}c_{31}=0$. So $c_{12}=0$ and then by Eq. (96), $c_{22}=0$. By Eq. (94), we have $c_{11}+c_{33}=0.$ Then Eq. (100) gives $c_{33}=\pm \sqrt{ac}i$. Thus $c_{11}=\mp \sqrt{ac}i$. By Eq. (97), we have $c_{21}=-\frac{c_{23}c_{33}}{c_{13}}.$ So $c_{21}=\mp \frac{d\sqrt{c}i}{\sqrt{a}}$. Then we obtain the solutions

$$N_{5,5}=\left(\begin{array}{ccc} -\sqrt{ac}i & 0 & a\\ -\frac{d\sqrt{c}i}{\sqrt{a}} & 0 & d\\ c & 0 & \sqrt{ac}i \end{array}\right)\;\text{and}\;N_{5,6}=\left(\begin{array}{ccc} \sqrt{ac}i & 0 & a\\ \frac{d\sqrt{c}i}{\sqrt{a}} & 0 & d\\ c & 0 & -\sqrt{ac}i \end{array}\right)\;(a,b,c,d\in k,a,c,d\neq 0).$$

**Subcase 3.** *c* = 0 and a,b,d≠0: Then By Eq. (98), we have $c_{32}c_{21}=0$. So $c_{21}=0$. By Eq. (92), we have $c_{11}=0$. Thus Eq. (99) gives $c_{22}+c_{33}=0$ and Eq. (100) gives $c_{33}=\pm \sqrt{bd}i$. Thus $c_{22}=\mp \sqrt{bd}i$. By Eq. (94), we have $c_{12}=-\frac{c_{13}c_{33}}{c_{23}}=\mp \frac{a\sqrt{b}i}{\sqrt{d}}$. Then we obtain the solutions

$$N_{5,7}=\left(\begin{array}{ccc} 0 & -\frac{a\sqrt{b}i}{\sqrt{d}} & a\\ 0 & -\sqrt{bd}i & d\\ 0 & b & \sqrt{bd}i \end{array}\right)\;\text{and}\;N_{5,8}=\left(\begin{array}{ccc} 0 & \frac{a\sqrt{b}i}{\sqrt{d}} & a\\ 0 & \sqrt{bd}i & d\\ 0 & b & -\sqrt{bd}i \end{array}\right)\;(a,b,c,d\in k,a,b,d\neq 0).$$

**Subcase 4.** *d* = 0 and a,b,c≠0: Then by Eq. (97), we have $c_{21}c_{13}=0$. So $c_{21}=0$. Thus by Eq. (96), we have $c_{22}=0$. So Eq. (94) gives $c_{11}+c_{33}=0$. Furthermore, by Eq. (100), we have $c_{33}=\pm \sqrt{ac}i$. Thus $c_{11}=\mp \sqrt{ac}i$. By Eq. (99), we get $c_{12}=-\frac{c_{32}c_{33}}{c_{31}}=\mp \frac{b\sqrt{a}i}{\sqrt{c}}$. Then we obtain the solutions

$$N_{5,9}=\left(\begin{array}{ccc} -\sqrt{ac}i & -\frac{b\sqrt{a}i}{\sqrt{c}} & a\\ 0 & 0 & 0\\ c & b & \sqrt{ac}i \end{array}\right)\;\text{and}\;N_{5,10}=\left(\begin{array}{ccc} \sqrt{ac}i & \frac{b\sqrt{a}i}{\sqrt{c}} & a\\ 0 & 0 & 0\\ c & b & -\sqrt{ac}i \end{array}\right)\;(a,b,c,d\in k,a,b,c\neq 0).$$

**Case 3.** Exactly two of a,b,c,d are 0: There are six subcases to consider. But note that if a=b=0,c≠0 and d≠0, i.e. $c_{13}=c_{32}=0,c_{31}\neq 0$ and $c_{23}\neq 0$, then by Eq. (99), we have $c_{12}c_{31}=0$. So $c_{12}=0$. Thus Eqs. (92) and (96) give $c_{11}=0$ and $c_{22}=0$, respectively. Then by Eq. (95), we have $c_{23}c_{31}=cd=0$, a contradiction. Similarly, if c=d=0 and a,b≠0, then we can obtain $c_{13}c_{32}=ab=0$, a contradiction. So there are four subcases left to consider.

**Subcase 1.** a=c=0 and b,d≠0: Then $c_{13}=c_{31}=0,c_{32}\neq 0$ and $c_{23}\neq 0$. Thus by Eq. (94), we have $c_{12}c_{23}=dc_{12}=0$. So $c_{12}=0$. Thus by Eq. (92), we have $c_{11}=0$. Then Eq. (99) gives $c_{22}+c_{33}=0$. By Eq. (100), we have $c_{33}=\pm \sqrt{bd}i$. Thus we get $c_{22}=\mp \sqrt{bd}i$. Then we get the solutions

$$N_{5,11}=\left(\begin{array}{ccc} 0 & 0 & 0\\ 0 & \sqrt{bd}i & d\\ 0 & b & -\sqrt{bd}i \end{array}\right)\text{and}N_{5,12}=\left(\begin{array}{ccc} 0 & 0 & 0\\ 0 & -\sqrt{bd}i & d\\ 0 & b & \sqrt{bd}i \end{array}\right)\;(b,d\in k\setminus \{0\}).$$

**Subcase 2.** a=d=0 and b,c≠0: Then $c_{13}=c_{23}=0,c_{32}\neq 0$ and $c_{31}\neq 0$. Eq. (100) gives $c_{33}=0$. Then by Eq. (98), we obtain $c_{11}=-\frac{c_{32}}{c_{31}}c_{21}=-\frac{b}{c}c_{21}$. From Eq. (99), we have $c_{12}=-\frac{c_{32}}{c_{31}}c_{22}=-\frac{b}{c}c_{22}$.

(1) If $c_{21}=0$, then $c_{11}=0$. By Eq. (96) we have $c_{22}=0$. So $c_{12}=0$. Then we obtain the solutions

$$N_{5,13_{1}}=\left(\begin{array}{ccc} 0 & 0 & 0\\ 0 & 0 & 0\\ c & b & 0 \end{array}\right)\;(b,c\in k\setminus \{0\}).$$

(2) If $c_{21}\neq 0$, then denote $e=c_{21}$. Thus by $c_{11}=-\frac{b}{c}c_{21}$, we have $c_{11}=-\frac{be}{c}$. By Eq. (95), we have $c_{11}+c_{22}=0$. Thus $c_{22}=\frac{be}{c}$. So we have $c_{12}=-\frac{b}{c}c_{22}=-\frac{b^{2}e}{c^{2}}$. Then we obtain the solutions

$$N_{5,13_{2}}=\left(\begin{array}{ccc} -\frac{be}{c} & -\frac{b^{2}e}{c^{2}} & 0\\ e & \frac{be}{c} & 0\\ c & b & 0 \end{array}\right)\;(b,c,e\in k\setminus \{0\}).$$

In summary, we obtain the solutions

$$N_{5,13}=\left(\begin{array}{ccc} -\frac{be}{c} & -\frac{b^{2}e}{c^{2}} & 0\\ e & \frac{be}{c} & 0\\ c & b & 0 \end{array}\right)\;(b,c\in k\setminus \{0\},e\in k).$$

**Subcase 3.** b=c=0 and a,d≠0: This subcase is similar to the above Subcase 2. Denote $e=c_{21}$. Then we can obtain the solutions

$$N_{5,14}=\left(\begin{array}{ccc} -\frac{ae}{d} & -\frac{a^{2}e}{d^{2}} & a\\ e & -\frac{ae}{d} & d\\ 0 & 0 & 0 \end{array}\right)\;(a,d,e\in k,a,d\neq 0).$$

**Subcase 4.** b=d=0 and a,c≠0: By Eq. (100), $c_{33}=\pm \sqrt{ac}i$. Then by Eq. (94), we have $c_{11}=\mp \sqrt{ac}i$. Then we can obtain the solutions

$$N_{5,15}=\left(\begin{array}{ccc} -\sqrt{ac}i & 0 & a\\ 0 & 0 & 0\\ c & 0 & \sqrt{ac}i \end{array}\right)\text{and}N_{5,16}=\left(\begin{array}{ccc} \sqrt{ac}i & 0 & a\\ 0 & 0 & 0\\ c & 0 & -\sqrt{ac}i \end{array}\right)\;(a,c\in k\setminus \{0\}).$$

**Case 4.** Exactly three of a,b,c,d are 0: Then we divide into four subcases to consider.

**Subcase 1.** a=b=c=0 and d≠0: Then $c_{13}=c_{32}=c_{31}=0$ and $c_{23}\neq 0$. Thus by Eq. (94), we have $c_{12}c_{23}=0$. So $c_{12}=0$ and then Eqs. (92) and (96) give $c_{11}=c_{22}=0$. By Eq. (100), we have $c_{33}=0$. Denote $e=c_{21}$. Then we obtain the solutions

$$N_{5,17}=\left(\begin{array}{ccc} 0 & 0 & 0\\ e & 0 & d\\ 0 & 0 & 0 \end{array}\right)\;(d,e\in k,d\neq 0).$$

Similarly, we obtain the following solutions for the rest of the subcases.

**Subcase 2.** a=b=d=0 and c≠0: Denote $e=c_{21}$. Then we have

$$N_{5,18}=\left(\begin{array}{ccc} 0 & 0 & 0\\ e & 0 & 0\\ c & 0 & 0 \end{array}\right)\;(c,e\in k,c\neq 0).$$

**Subcase 3.** a=c=d=0 and b≠0: Denote $e=c_{12}$. Then we have

$$N_{5,19}=\left(\begin{array}{ccc} 0 & e & 0\\ 0 & 0 & 0\\ 0 & b & 0 \end{array}\right)\;(b,e\in k,b\neq 0).$$

**Subcase 4.** b=c=d=0 and a≠0: Denote $e=c_{12}$, where e∈k. Then we have

$$N_{5,20}=\left(\begin{array}{ccc} 0 & e & a\\ 0 & 0 & 0\\ 0 & 0 & 0 \end{array}\right)\;(a,e\in k,a\neq 0).$$

**Case 5.** a=b=c=d=0: By Eq. (100), we have $c_{33}=0$. Denote $e=c_{12}$ and $f=c_{21}$. Then by Eqs. (92) and (96), we have $c_{11}=\pm \sqrt{ef}i$ and $c_{22}=\mp \sqrt{ef}i$.

(1) If $c_{12}\neq 0$ or $c_{21}\neq 0$, by Eqs. (93) and (95), we have $c_{11}+c_{22}=0$. Then we can obtain the solutions

$$N_{5,21_{1}}=\left(\begin{array}{ccc} \sqrt{ef}i & e & 0\\ f & -\sqrt{ef}i & 0\\ 0 & 0 & 0 \end{array}\right)\;\text{and}\;N_{5,21_{2}}=\left(\begin{array}{ccc} -\sqrt{ef}i & e & 0\\ f & \sqrt{ef}i & 0\\ 0 & 0 & 0 \end{array}\right)\;(e\neq 0\text{or}f\neq 0)$$

(2) If $c_{12}=c_{21}=0$, then by Eqs. (92) and (96), we have $c_{11}=c_{12}=0$. Thus we obtain the zero solution $N_{5,21_{3}}=0_{3\times 3}.$

In summary, we obtain the solutions

$$N_{5,21}=\left(\begin{array}{ccc} \sqrt{ef}i & e & 0\\ f & -\sqrt{ef}i & 0\\ 0 & 0 & 0 \end{array}\right)\;\text{and}\;N_{5,22}=\left(\begin{array}{ccc} -\sqrt{ef}i & e & 0\\ f & \sqrt{ef}i & 0\\ 0 & 0 & 0 \end{array}\right)\;(e,f\in k).$$

This completes the proof of the case for k[NCS(5)].

With the remark made in Section 5.2.2, now the proof of Theorem 5.1 is completed.

## 6. Conclusion

We have presented a complete and explicit classification of Rota-Baxter operators on semigroup algebras for the orders 2 and 3. With some care taken to ensure efficient calculations, the same approach could be used for classifying all semigroup algebras over semigroups of order 4. This would provide a valuable stock of “finite” exemplary objects in the Rota-Baxter category. It would also be interesting to explore Rota-Baxter structures on other classes of algebras, e.g. low-dimensional path algebras, matrix rings, and special types of group algebras. As an example for the latter, consider cyclic groups: By the results presented above we know that all Rota-Baxter operators over the cyclic group of order 2 or 3 are trivial—is this true for any (prime order) cyclic group?
